# The Choice of an Antiseptic

**Published:** 1892-11-12

**Authors:** 


### NORTHAMPTON GENERAL INFIRMARY.

### The Choice of an Antiseptic.

The dressing of a clean-cut wound in a country district, amongst healthy and pure surroundings, is a most simple matter, and our country colleagues are inclined to ridicule the care which hospital surgeons invariably exercise in dressing their wounds. A little Friar's balsam or dry lint binding up the wound with the blood so as to form an artificial scab is, in their opinion, all that is necessary, and with their surroundings, it probably is so. But the case is very different in hospital practice, where every precaution must be taken, under necessarily much worse surroundings, to prevent the entrance into the building of any septic disease which may spread to other cases. Every good surgeon will prefer to take all precautions that may suggest themselves to guard his patient from any lurking danger, even in the most trivial case. The great improvement in surgical dressings of late years has been brought about by the use of dry absorbent dressings. They are all most useful, whether dry dressings only are used, as Gamgee tissue and absorbent cotten wool, or some antiseptic is combined with the absorbent, as the various kinds of gauze and medicated cotton wools (carbolic, thymol, salicylic, sal alembroth, double cyanide, &c.), Hartman's wood wool, &c. To take a most simple case, as the dressing of an operation wound (excision of breast, let us say). After the breast has been removed and all hæmorrhage arrested, the following is the plan usually adopted at this hospital: Every corner of the wound is thoroughly swabbed out with an antiseptic lotion, a drainage tube is inserted through the lowest corner of the wound (not through the whole length of the wound, so as to act as a seton), then the stitches are inserted and tied. The surface of the wound and skin is again washed with more of the lotion, carefully dried, powdered with an antiseptic powder, and then several layers of one of the above absorbent dressings and cotton wool are carefully and firmly bandaged over it. In carrying out this plan two different sorts of antiseptics are required—a liquid and a powder—and it is to the selection of these antiseptics that this paper is devoted, for it is impossible to treat, save in a piecemeal fashion, the very large questions involved in the choice of surgical dressings. The following are the chief antiseptics in present use:

| As Liquids. | As Powders. |
|---|---|
| Carbolic acid (1 in 40). | Iodoform. |
| Corrosive sublimate (1 in 2,000), | Boracic acid. |
| Boracic acid (nearly saturated solution.) | Iodol. |
| Salufer (1 grain in 1 oz.) | Salol. |

A model antiseptic ought (quoting Professor Thompson) to fulfil the following conditions: (1) It should be efficient; (2) it should give rise to no irritation; (3) it should be non-poisonous; (4) it should be odourless and preferably colourless; (5) it should be non-volatile and should not become useless on exposure to air. Applying this test to the liquids on the above list it will be found that carbolic acid will hardly fulfil one of these requirements, though it is in such general use, and it has been answerable for a very large number of accidental deaths. Surely it is time this dangerous drug was consigned to oblivion. Corrosive sublimate is very efficient, but is so poisonous as to be out of court if anything as serviceable can be found to replace it, and boracic acid is not efficient unless used in very strong solution. Salufer is the only drug that fulfils all the conditions; it has been used here now for many years by one of the surgical staff, and it is strange that so useful an antiseptic is not in universal use, especially as other surgeons in Leeds and elsewhere have testified to its great utility. It is a white powder, dissolving only to the extent of 2 grains in 1 oz. of water, non-poisonous, and odourless. It is stated that the powder itself is an irritant, and must not be dusted on wounds, but no irritating effect has been observed here from its use in solution. It is generally kept in the wards as a saturated solution (2 grains in 1 oz.), and for use is diluted with equal parts of hot water; this strength is found quite efficient. Professor Thompson, who introduced it, found that its saturated solution preserved chopped meat for a longer time than a solution of corrosive sublimate (1 in 500), and that it preserved things from decomposition much longer than any known antiseptic. Its only drawback is that like corrosive sublimate it takes the polish off steel instruments, so that for an operation these should be kept immersed in some other disinfectant solution. For private practice it is very conveniently put up in cubes, one of which is sufficient for a quart of water, and the cubes are packed in small metal boxes about the size of a fusee box. Chemically salufer is known as silico-fluoride of sodium.

Next as to the choice of an antiseptic powder for dusting over a wound. Since the spray has been abandoned, the great idea in the surgical mind of the present day is to swamp any unpleasant smell that is likely to arise with a worse one. Hence, probably, the profuse and indiscriminate use of iodoform, than which a worse smell does not exist. The old practitioners who dispensed their own medicines were often sneered at as smelling of jalap and rhubarb, and now private patients sneer, and with reason, at their surgeons coming to them reeking with the smell of carbolic acid or iodoform. Surely this is not necessary. So abominable is the smell of iodoform in a private house to those unaccustomed to it that operations have, to the writer's knowledge, been declined unless some substitute could be employed. Surely, too, the use of drugs that will mask the warning sense of smell and thus prevent us from detecting the first sign of putridity about a wound is a real, and not an imaginary danger. Iodol is a good substitute for iodoform, but its colour is an objection, and it is very dear. Salol is very much used at this hospital with the best results; it is colourless, and has only a faint, not unpleasant smell. Boracic acid powder would probably be just as serviceable, and neither of these latter will cause so much irritation, especially to the skin, as does iodoform.

A German paper, giving the strength of various solutions necessary to destroy the micrococci of pus is not so generally known as it deserves to be, especially as its conclusions differ considerably from the views generally held. The following results bear chiefly on this paper: Iodine, 1 in 10,000; thymol, 1 in 5,000; liq. sodium hypochlorite, acid. nitric., and hydrarg. perchlor., 1 in 1,000; acid. salicylic., 1 in 300; acid carbolic and potass. permanganat., 1 per cent.; acid. boric, 4 per cent.; zinci chlorid, 5 per cent. The high position of iodine and thymol in this list should lead to a more extensive trial of these drugs than has heretofore been accorded them. In the requisite dilute solutions they would not be open to the same objections that have been raised to various antiseptics mentioned in this paper.

The whole question of antiseptics has not received that experimental observation which the importance of the subject demands. In making a few comparative experiments on the four quoted antiseptics the writer met with some curious results, but was unable, for many reasons, to continue the investigations. Certain substances were kept in a very warm, damp corner of a greenhouse, freely exposed to the air, and the above antiseptics in the strength ordinarily used were added. In the first instance a paste of flour and water was made, and the different antiseptics added to each separate quantity. A fungus was growing over the top of the boric acid mixture in seven days; in ten days it was quite bad. The carbolic acid mixture lasted four weeks, but was then dark and decomposed, whilst the salufer and corrosive sublimate mixtures kept quite good for six weeks, when they had dried up.

In the next experiment a mixture of minced meat and water was exposed alone, and was perfectly putrid and decomposed in three days. The other mixtures with each different solution all kept quite good and free from smell for six weeks; the salufer mixture only had a slight growth of fungus on it, but there was no offensive smell, and the boric acid was just commencing to smell a little at the end of that time.

Another experiment was made with equal parts of urine and the antiseptic. It agreed with the others in showing the surprising length of time this mixture could be kept compared with a test experiment of urine alone, and the very great value of each one of these antiseptics; but one agent seemed to stop one kind of putrefaction best and another another kind. The experiments were not carried out on a large enough scale to be really serviceable, but extended experiments on these lines might lead to some practical results.

